# The complete mitochondrial genome of *Linguatula serrata* (tongue worm) isolated from a dog and phylogenetic analysis

**DOI:** 10.1080/23802359.2018.1450679

**Published:** 2018-03-14

**Authors:** Tanian Naude, Sameer Pant, Mousa Tavassoli, Subir Sarker, Seyed Ali Ghorashi

**Affiliations:** a School of Animal and Veterinary Sciences, Charles Sturt University, Wagga Wagga, Australia;; b Graham Centre for Agricultural Innovation, Wagga Wagga, Australia;; c Faculty of Veterinary Medicine, Urmia University, Urmia, Iran;; d Department of Physiology, Anatomy and Microbiology, La Trobe University, Victoria, Australia

**Keywords:** *Linguatula serrata*, complete mitogenome, phylogenetic tree

## Abstract

The complete mitogenome of *Linguatula serrata* isolated from nasal cavity of a dog was characterized for the first time. The total size of the circular mitogenome was 15,328 bp consisting of 37 genes including 13 protein coding genes, 22 tRNA genes, two rRNA genes and two control regions. Phylogenetic tree was constructed based on 17 closely related species and their genetic relationship with *Linguatula serrata* was analysed.

*Linguatula serrata* is an aberrant arthropod of the class Pentastomida (Hendrix 1998). The adult parasite is found in the nasopharynx of canids (Khalil and Schacher 1965). The larval form develops to the infective nymphal stage in visceral organs of herbivores and canids can be infected by consuming uncooked infected visceral organs of the herbivorous hosts (Soulsby 1982). Linguatulosis is a zoonotic disease and has been reported from human cases from different parts of the world and particularly Middle East (Schacher et al. 1969; Sadjadi et al. 1998; Maleky 2001). Human beings can be infected by both the nymph stage and egg, which are called Halzoun syndrome and visceral linguatulosis, respectively (Lazo et al. 1999). Human infection may occur via consumption of raw or undercooked liver or visceral organs associated with lymph nodes of infected animals (Beaver 1984; Drabick 1987; el-Hassan et al. 1991).

Total genomic DNA was extracted from individual *Linguatula serrata* adult worm (Accession No. IR-17-Dog-Urmia) as described before (Ghorashi et al. 2016).

The complete mitochondrion genome was sequenced using Illumina sequencing and assembled using Novoplasty software (Dierckxsens et al. 2017). *Linguatula serrata* mitogenome was annotated using MITOS (Bernt et al. 2013) and has a circular mitochondrial genome of 15,328 bp containing 4797 Adenine, 4438 Thymine, 1016 Guanine, and 5077 Cytosine. The GC content was 39.8%. The total length of coding sequences was 9678 base pairs (63.1% of the total sequence). The lengths of 12S and 16S ribosomal RNA were 537 and 623 base pairs, respectively. The gene arrangement was similar to the complete mitochondrial DNA of *Armillifer agkistrodontis* (Li et al. 2016). The complete *Linguatula serrata* mitogenome has been deposited at the GenBank under accession no. MG951756.

Complete mitochondrion DNA sequences of 14 Arthropoda including two pentastomida (*Speleonectes tulumensis* (AY456190), *Armillifer armillatus* (AY456186), *Armillifer agkistrodontis* (KX686568), *Abacion magnum* (JX437062), *Nobia grandis* (KF720334), *Lepas australis* (KM017964), *Pollicipes polymerus* (AY456188), *Squilla empusa* (DQ191684), *Scutigerella causeyae* (DQ666065), *Rhipicephalus microplus* (KP143546), *Ixodes ricinus* (KF197115), *Ammothea carolinensis* (GU065293), *Hutchinsoniella macracantha* (AY456189), *Argulus americanus* (AY456187)) and three Nematoda (*Trichinella trichuria* (GU385218), *Trichinella spiralis* (AF293969), *Trichuris ovis* (JQ996232)) available in the GenBank, were retrieved and aligned with draft *Linguatula serrata* sequence. The *Fasciola gigantica* (KF543342) complete mitochondrion genome (Trematode) was included as out group. The sequences were analysed using ClustalW (Thompson et al. 1994) within DNASTAR software and BioEdit Sequence Alignment Editor (version 6.0.9.0). Multiple sequence alignment was generated using a gap open penalty of 10 and gap extension penalty of 1. The phylogenetic tree was generated using the maximum likelihood (ML) method. The phylogeny was carried out with the bootstrap method using 1000 bootstrap replications.

The pentastomida (tongue worms) were genetically closer to Nematods compared to Arthropods. Among the three pentastomida, *Armillifer armillatus* and *Armillifer agkistrodontis* were more closely related when compared to *Linguatula serrata* (Figure 1). The complete mitogenome of *Linguatula serrata* could potentially assist in proper classification of the parasite, and in the development of DNA based genotyping and diagnostics.

**Figure 1.**
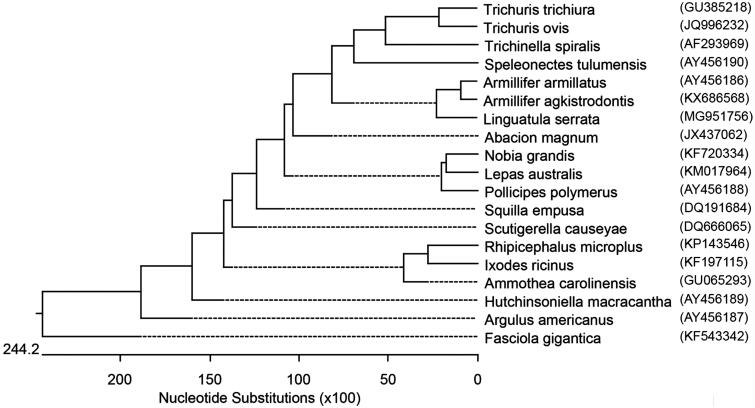
Phylogenetic tree generated by Clustal W software. *Linguatula serrata* has been placed into the class of maxillopoda formed a sister clade to the pentastomids *Armillifer agkistrodontis* and *Armillifer armillatus*.
